# Reproducible measurable residual disease detection by multiparametric flow cytometry in acute myeloid leukemia

**DOI:** 10.1038/s41375-022-01647-5

**Published:** 2022-07-18

**Authors:** Maximilian A. Röhnert, Michael Kramer, Jonas Schadt, Philipp Ensel, Christian Thiede, Stefan W. Krause, Veit Bücklein, Jörg Hoffmann, Sonia Jaramillo, Richard F. Schlenk, Christoph Röllig, Martin Bornhäuser, Nicholas McCarthy, Sylvie Freeman, Uta Oelschlägel, Malte von Bonin

**Affiliations:** 1 Department of Medicine I, University Hospital Carl Gustav Carus TU Dresden, Dresden, Germany; 2 AgenDix GmbH, Dresden, Germany; 3 Department of Medicine 5, Universitätsklinikum Erlangen, Erlangen, Germany; 4 Department of Medicine III, University Hospital LMU Munich, Munich, Germany; 5 Laboratory for Translational Cancer Immunology, Gene Center, LMU Munich, Munich, Germany; 6 Department of Internal Medicine and Hematology, Oncology and Immunology, Philipps University Marburg and University Hospital Giessen and Marburg, Marburg, Germany; 7 Department of Internal Medicine V, Heidelberg University Hospital, Heidelberg, Germany; 8 NCT Trial Center, National Center of Tumor Diseases, German Cancer Research Center, Heidelberg, Germany; 9 National Center of Tumor Diseases, Dresden, Germany; 10 Institute of Immunology and Immunotherapy, University of Birmingham, Birmingham, UK

**Keywords:** Acute myeloid leukaemia, Acute myeloid leukaemia

## Abstract

Measurable residual disease (MRD) detected by multiparametric flow cytometry (MFC) is associated with unfavorable outcome in patients with AML. A simple, broadly applicable eight-color panel was implemented and analyzed utilizing a hierarchical gating strategy with fixed gates to develop a clear-cut LAIP-based DfN approach. In total, 32 subpopulations with aberrant phenotypes with/without expression of markers of immaturity were monitored in 246 AML patients after completion of induction chemotherapy. Reference values were established utilizing 90 leukemia-free controls. Overall, 73% of patients achieved a response by cytomorphology. In responders, the overall survival was shorter for MRD^pos^ patients (HR 3.8, *p* = 0.006). Overall survival of MRD^neg^ non-responders was comparable to MRD^neg^ responders. The inter-rater-reliability for MRD detection was high with a Krippendorffs α of 0.860. The mean time requirement for MRD analyses at follow-up was very short with 04:31 minutes. The proposed one-tube MFC approach for detection of MRD allows a high level of standardization leading to a promising inter-observer-reliability with a fast turnover. MRD defined by this strategy provides relevant prognostic information and establishes aberrancies outside of cell populations with markers of immaturity as an independent risk feature. Our results imply that this strategy may provide the base for multicentric immunophenotypic MRD assessment.

## Introduction

Acute myeloid leukemia (AML) is a heterogeneous disease. After undergoing intensive induction chemotherapy, about 70% of eligible patients achieve a complete remission (CR). Without further treatment, 50% of the patients relapse within 6 months [1]. Post induction therapy is stratified by relapse risk and includes chemotherapy or allogeneic hematopoietic stem cell transplantation (aHSCT). The prognosis is partially determined upfront by cytogenetic and molecular genetic aberrations [2]. Remaining leukemic cells in bone marrow (BM) with <5% blasts are called measurable residual disease (MRD) and provide additive information for tailored treatment decisions and refinement of the prognosis. MRD positivity indicates residual disease and a high probability of relapse, whereas MRD negativity characterizes deep CR with low risk of relapse. At diagnosis, in at least 80% of AML patients molecular genetic aberrations are detectable by next-generation sequencing [3]. However, only some of these aberrations can be detected with sensitive routine assays providing clinically relevant prognostic information: m*NPM1* is present in 30% [4], *CBFB::MYH11* in 5% [5] and *RUNX1::RUNX1T1* in 7% [2, 5–7] of non-acute promyelocytic leukemia patients, respectively.

MRD monitoring by multiparametric flow cytometry (MFC) has been shown to be applicable to almost all patients [8–17]. The precise workflow varies across laboratories. Sample processing and measurement differ between institutions, and there are two distinct analysis strategies to detect leukemic cell populations: the leukemia-associated immunophenotype (LAIP) and the different from normal (DfN) approach. In the LAIP concept, one or more individual LAIP are identified at diagnosis and tracked during follow-up. Depending on the antibody panel, at least one LAIP with aberrant antigen expression pattern is found in 80–95% of patients at diagnosis [18, 19]. The DfN strategy searches for aberrant immunophenotypes rarely observed in leukemia-free BM during follow up independent of pre-treatment samples [20]. Both strategies require experienced investigators and rely partly on individualized gates. The LAIP approach is considered more sensitive, but also more susceptible to phenotypic shifts and false negative results [21, 22]. The LAIP analysis is time consuming and the individualized gating for each patient leads to a low inter-rater reliability (IRR) [18, 23, 24]. In contrast, the DfN approach may lead to false positive results due to reactive changes in hematopoiesis exposed to chemotherapy [25, 26]. In addition, differences in the analysis strategy for both concepts lead to heterogeneous MRD results with different prediction values [27, 28]. As LAIP and DfN approaches differ in their strengths and weaknesses, the European LeukemiaNet (ELN) recommends a combinatorial concept, termed LAIP-based DfN approach [29].

The HARMONIZE consortium was established in 2016 to implement standards for MFC based MRD detection within two German AML study groups (SAL, AMLCG). Here, we present a stable, fast and reproducible LAIP-based DfN analysis approach that preserves the prognostic value of MRD assessment.

## Methods

### Study cohort

Samples from newly diagnosed AML patients at diagnosis (in median 2 days *before* start of induction therapy) and after completion of intensive induction chemotherapy (in median 34 days *after* start of the last induction cycle) were shipped from 30 centers within 24–48 h to the laboratory of the AML Registry of the Study Alliance Leukemia (SAL) in Dresden (institutional review board Dresden, 98032010; clinicaltrials.gov, NCT03188874). Both, BM aspirates and peripheral blood were suitable for MFC at diagnosis. At follow-up, only BM aspirates were utilized. The clinical data was extracted from the registry.

### Reference cohort

All leukemia-free controls (LFC, *n* = 90) were treated at the University Hospital Dresden (Supplementary Table 1) and analyzed by three independent investigators. The aberrant subpopulations as described below (*n* = 32) were also observed in LFC with different frequencies. The upper limit of the one-sided 97.5% reference range for the percentage of each aberrant population among CD45^+^ events was set as reference value. LFC included BM aspirates of BM donors (*n* = 30), of patients with acute lymphoblastic leukemia in molecular CR (ALL molCR) (*n* = 19) with a prior exposure to chemotherapy (median 40 days *after* start of the last chemotherapy cycle), of hip surgery patients (*n* = 32) representing older patients and of patients with untreated primary central nervous system lymphoma (PCNSL, *n* = 9).

### Sample preparation and acquisition

The antibody panel (Supplementary Table 2) consists of eight monoclonal antibodies (mAb) and was designed in 2016 by the HARMONIZE consortium [30–32]. The ELN also recommends the targeted antigens as mandatory core MRD markers [33]. In addition, a comparable mAb panel is used by HOVON/SAKK [29, 34, 35]. However, both panels propagate different mAb clones with divergent fluorochromes.

At least 500,000 events were acquired per tube. The samples were measured centrally and analyzed by at least one of three different investigators. Further details concerning cell preparation and acquisition can be found in the Supplementary material.

### Gating strategy

Kaluza 2.1 software (Beckman Coulter) was used for analysis. An SQL server and Excel (all by Microsoft) served to analyze, store and process the data.

Our proposed LAIP-based DfN analysis is based on a hierarchical gating strategy with fixed gates. The investigators adjusted only the gates for doublet discrimination, exclusion of debris, leukocytes, progenitors/monocytes (P/M) and lymphocytes. The P/M population had to express at least one of the myeloid markers CD13 or CD33 (myP/M) and was subdivided afterward by expression of CD34, CD117 and HLA-DR (the backbone markers recommended by the EuroFlow Consortium) [36] resulting in 8 myP/M main populations. These main populations were further characterized by four distinct aberrant categories: deficiency of CD13 or CD33, cross-lineage expression of CD7 or CD56 leading to 32 subpopulations. Subpopulations that exceeded their reference values were used to calculate the MRD load. The difference between measurement and reference value represented disease burden (percentage of CD45^+^ cells). Disease burden of subpopulations with identical aberrant category were summed up leading to an aggregated size for deficiency of CD13 or CD33, cross-lineage expression of CD7 or CD56. Only the aberrant category with the highest sum was used to quantify the MRD load.

**Fig. 1:**
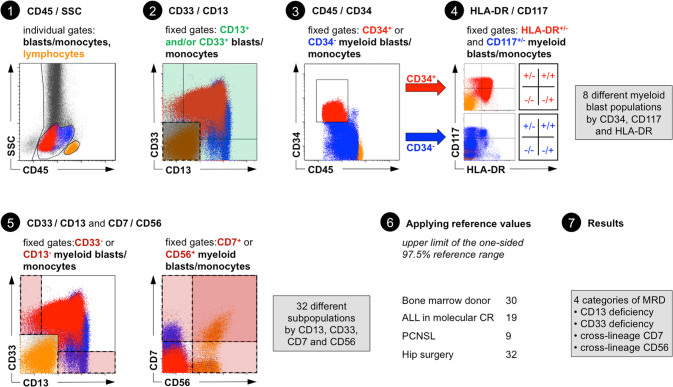
Detailed gating strategy for the proposed LAIP-based DfN MRD approach. (1) individual gating for blasts/monocytes and lymphocytes using CD45/SSC (2) fixed gates for the expression of at least one myeloid marker (CD13 and/or CD33) on events in the blasts/monocytes gate (3) fixed gate for CD34 on myeloid blasts/monocytes (4) fixed gates to distinguish 8 different myeloid blast populations by CD34, CD117 and HLA-DR (5) fixed gates to define events within the myeloid blasts/monocytes with a deficiency of CD13 or CD33 and a cross-lineage expression of CD7 or CD56 leading to 32 subpopulations (6) applying reference values for each subpopulations (7) MRD assessment by four different categories. Not shown: exclusion of doublets and debris.

In our approach, the gates for the myeloid markers (CD13, CD33), the backbone markers (CD34, CD117 and HLA-DR) and the cross-lineage markers (CD7, CD56) were fixed (Fig. 1). The positioning of the fixed gates was driven by review of reference measurements (leukemia-free controls) and internal controls within AML samples (in particular lymphocytes). The leukemic cell population itself never guided the definition of the fixed gates. Consequently, gates sometimes cut through leukemic cell populations and did not follow visual demarcations within the blast population. The gates in our strategy identify pronounced aberrant features only. Deficiency of CD13 and CD33 represent a true absence of these antigens rather than a weak expression. The cross-lineage expression of CD7 and CD56 represents a strong rather than a weak expression.

### Definition of MRD in AML samples

At diagnosis, an aberrant category was defined as LAIP (aLAIP) when ≥10% myP/M were affected. At follow-up only subpopulations with at least ≥20 events were analyzed. MRD^pos^ was defined by at least one aberrant subpopulation exceeding its reference value. When this aberrant category was already detectable at diagnosis this reoccurrence was defined as MRD^pos^ by aLAIP. MRD^pos^ by post-treatment DfN (ptDfN) was defined as de-novo appearance of an aberrant category.

As ELN recommends a flat 0.1% cut-off for MRD [2, 33], we interpreted our data in two additional ways. First, the analysis was restricted to aberrant subpopulations expressing at least one marker of immaturity and the cut-off for those populations was uniformly set to 0.1% (MRD^posELN^ vs. MRD^negELN^). Second, MRD^pos^ was subdivided according to the MRD load in MRD^posLo^ (our reference values were exceeded by <0.1) and MRD^posHi^ (excess of the reference values by ≥0.1).

Many MFC approaches rely on populations expressing markers of immaturity (e.g. CD34^+^ and/or CD117^+^) whereas monopoietic cells (identified by SSC and CD45) are frequently excluded. Monocytic AMLs often present without immunophenotypically immature populations [37]. Therefore, we performed additional analyses selecting only aberrant subpopulations expressing at least one marker of immaturity (*n* = 24, MRD^posImmOnly^ vs. MRD^negImmOnly^).

### Cross-validation

To validate the proposed MRD approach, the results were compared with two already published and established methods to analyze MFC data for presence of MRD: a traditional (manual) flow cytometry approach based on the conventional detection of an aberrant LAIP (convLAIP) [19, 38] and an unsupervised computational approach (Unsup) [39, 40]. The convLAIP approach was restricted to the samples used to calculate the IRR of the proposed approach (*n* = 117, see below). Three investigators independently analyzed these samples. The Unsup approach encompassed all follow-up samples.

Furthermore, we compared the results of the proposed MRD approach also with molecular MRD results. Established and decisive molecular markers (*CBFB::MYH11*, m*NPM1* and *RUNX1::RUNX1T1*) as well as other clonal aberrations (e.g. m*RUNX1*, m*IDH1*) were used. A cut-off of 0.1% variant allele frequency was utilized as recommended recently by ELN to distinguish MRD positivity (Mol^pos^) and MRD negativity (Mol^neg^) [33].

Particular attention was given to patients rated MRD^pos^ only by ptDfN as this cohort was regarded vulnerable to misinterpretation.

### Inter-rater reliability (IRR)

Three independent investigators analyzed the first 117 follow-up and all LFC samples to define the IRR as quality parameter of the proposed MRD approach. Krippendorffs α (Kα) as value for the IRR was calculated for two parameters: (I) percentage of CD45^+^ events for each of the 32 subpopulations within the LFC and (II) the final MRD status within the AML samples.

### Time requirements for sample analysis

The time to perform the different analysis steps was independently evaluated in 10 samples of the LFC and 10 samples of patients with AML at diagnosis and at follow-up by three investigators, respectively. Different work steps were evaluated: (I - gating) Import of MFC files into Kaluza software and adjusting the non-fixed gates; (II - export) Export of raw data into the SQL database; (III – report) evaluating the MRD status using Excel and creating a MRD report using an Access database.

### Statistical analysis

To define IRR, Kα, a reliability coefficient ranging from 0 to 1, with 1 representing perfect agreement between multiple raters [41], was calculated.

To quantitatively compare different models in their ability to predict clinical outcome, the Akaike information criterion (AIC) was used [42, 43].

The Kaplan–Meier method was used to estimate survival probabilities. Survival curves were compared utilizing the Cox regression model. Multivariable Cox regression models were used to describe the effect of different variables on survival. A *p* < 0.05 was regarded as statistically significant. Overall survival (OS) was defined as the time from diagnosis to death from all causes, relapse free survival (RFS) as the time from response to AML relapse or death. In this regard, response was characterized by achievement of complete remission (CR), CR with incomplete hematologic recovery (CRi), or morphologic leukemia-free state (MLFS) [2]. Hematologic relapse, molecular relapse (2 consecutive positive samples for *NPM1mut/ABL* > 1% in a previously for *mNPM1* MRD negative patient) or a drop in overall chimerism <80% after aHSCT were consistent with relapse [44]. Event free survival (EFS) was defined as time from diagnosis to death from any cause, relapse or allogeneic hematopoietic stem cell transplantation >180 days after completion of intensive induction therapy, whatever occurred first.

## Results

### Reference values

Reference values were in the range of 0.001% of CD45^+^ for the aberrant subpopulation CD34^+^CD117^+^HLA-DR^-^CD56^+^ up to 1.992% for the subpopulation CD34^-^CD117^-^HLA-DR^+^CD13^-^. They were substantially influenced by the heterogeneity of the LFC cohorts. Fifteen of the 32 aberrant subpopulations (47%) were mainly influenced by ALL in molecular CR, 10 (31%) by BMD, 5 (16%) by patients undergoing hip surgery and only 2 (6%) by PCNSL (Supplementary Table 3). E.g. cross-lineage expression of CD56 and deficiency of CD13 were mostly seen in ALL, while CD33 deficiency was observed in patients undergoing hip surgery. In general, ALL samples showed the largest variance for most aberrant categories. Due to the minimum population size (≥20 events for AML samples), an aberrant subpopulation with a very low reference value can turn MRD positive (MRD^pos^) only when a large number of CD45^+^ events is acquired. For example, an aberrant subpopulation barely exceeding its reference value of 0.001%, at least 2,000,000 CD45^+^ events would be necessary to obtain ≥20 relevant events. Four aberrant subpopulations (CD34^+^CD117^+^HLA-DR^-^CD13^-^, CD34^+^CD117^+^HLA-DR^-^CD7^+^, CD34^+^CD117^+^HLA-DR^-^CD56^+^, CD34^+^CD117^-^HLA-DR^-^CD56^+^) were affected by this phenomenon at the targeted acquisition of 500,000 events.

### Inter-rater reliability (IRR) of the leukemia-free controls (LFC)

In the LFC cohort, Kα for the 8 main populations was 0.757–0.990. All but one main population presented with Kα ≥ 0.800. The populations with the lowest contingency (CD34^-^CD117^+^) only differed in the expression of HLA-DR. Even for the subpopulations with aberrant features (*n* = 32), there was a considerable high IRR for the percentage of CD45^+^ events with a Kα > 0.900 for the deficiency of CD13, followed by Kα for the deficiency of CD33, the cross-lineage expression of CD7 and the cross-lineage expression of CD56 with >0.800, >0.700, and >0.600, respectively (Supplementary Table 3).

### Time requirements for sample analysis

The mean time for gating (I) and export of the results (II) was 01:18 min and 00:47 min, without significant differences between diagnosis, follow-up and LFC samples. Overall time for analysis, data transfer and generation of a report was 04:17 min and 04:31 min for diagnosis and follow-up samples, respectively (Table 1).

**Table 1:** Time requirement (minutes:seconds) of the working steps at diagnosis and follow-up, leukemia-free controls (LFC).

	I - gating	II - export	III – report	overall
	Mean (SD)	Mean (SD)	Mean (SD)	Mean (SD)
Diagnosis	01:03 (00:12)	00:46 (00:09)	02:33 (00:16)	04:17 (00:33)
Follow-up	01:31 (00:30)	00:51 (00:11)	02:09 (00:20)	04:31 (00:49)
LFC	01:20 (00:17)	00:45 (00:08)	not intended	not intended
overall	01:18 (00:24)	00:47 (00:10)		

^a^*SD* standard deviation, *LFC* leukemia-free controls.

### MRD assessment

Our analysis included 246 patients with AML (non-APL) who were treated with intensive induction therapy and for whom reliable clinical data and at least one suitable MRD analysis at follow-up was available (Table 2).

**Table 2:** Patient and disease characteristics, MRD classification according to the proposed MRD approach.

		**Overall**	**MRD**^**neg**^	**MRD**^**pos**^	
		*n* = 246	*n* = 89 (36%)	*n* = 157 (64%)	
Age					
Median (IQR)	Years	56 (46–63)	50 (36–60)	58 (51–64)	*p* < 0.0001
>65 years	*n* (%)	36 (15)	7 (8)	29 (19)	*p* = 0.0381
Sex					
Female	*n* (%)	104 (42)	40 (45)	64 (41)	*p* = 0.6438
ECOG					
Miss	*n*	29	15	14	
0–1	*n* (%)	202 (93)	70 (95)	132 (92)	*p* = 0.7284
2–4	*n* (%)	15 (7)	4 (5)	11 (8)	
AML type					
Miss	*n*	27	13	14	*p* = 0.1783
De-novo	*n* (%)	185 (75)	68 (76)	117 (75)	
sAML	*n* (%)	19 (8)	6 (7)	13 (8)	
tAML	*n* (%)	15 (6)	2 (2)	13 (8)	
BM blasts in %					
Miss	*n*	28	14	14	
Median (IQR)		60 (39–80)	65 (50–80)	55 (33–80)	*p* = 0.1022
WBC in GPt/l					
Miss	*n*	27	13	14	
Median (IQR)		9.7 (2.9–43.8)	17.9 (4.3–43.6)	7.6 (2.7–43.3)	*p* = 0.0870
Complex karyotype					
Miss	*n*	50	19 (21)	31 (20)	
Yes	*n* (%)	15 (8)	1 (1)	14 (11)	*p* = 0.0306
*FLT3*					
Miss	*n*	12	7	5	
* FLT3*-ITD	*n* (%)	64 (27)	32 (39)	32 (21)	*p* = 0.0053
*NPM1*					
Miss	*n*	11	5	6	
* NPM1*^mut^	*n* (%)	83 (35)	36 (43)	47 (31)	*p* = 0.0967
ELN 2017					
Miss	*n*	4	0	4	
Favorable	*n* (%)	86 (36)	44 (49)	42 (28)	*p* = 0.0005
Intermediate	*n* (%)	89 (37)	31 (35)	58 (38)	
Adverse	*n* (%)	67 (28)	14 (16)	53 (35)	
MRC index					
Miss	*n*	55	21	34	
Good	*n* (%)	12 (6)	9 (13)	3 (2)	*p* < 0.0001
Standard	*n* (%)	73 (38)	34 (50)	39 (32)	
Poor	*n* (%)	106 (56)	25 (37)	81 (66)	
Induction					
2 cycles	*n* (%)	128 (52)	52 (58)	76 (48)	*p* = 0.1680
Consolidation					
Chemo	*n* (%)	76 (31)	40 (45)	36 (23)	*p* < 0.0001
alloHSCT <6 month	*n* (%)	107 (43)	34 (38)	73 (46)	
alloHSCT >6 month	*n* (%)	63 (26)	15 (17)	48 (31)	
Response at follow-up					
RD	*n* (%)	23 (9)	2 (2)	21 (13)	*p* < 0.0001
PR	*n* (%)	43 (18)	6 (7)	37 (24)	
CR/CRi/MLFS	*n* (%)	180 (73)	81 (91)	99 (63)	

^a^*IQR* interquartile range, *ECOG* Eastern Cooperative Oncology Group Performance Status Scale, *sAML* secondary AML, *tAML* therapy related AML, *BM* bone marrow, *WBC* white blood cell, *ELN 2017* European LeukemiaNet risk stratification by genetics 2017, *MRC Index* Medical Research Council Index for risk stratification of AML, *alloHSCT* allogeneic hematopoietic stem cell transplantation, *RD* refractory disease, *PR* partial remission, *CR* complete remission, *CRi* complete remission with incomplete count recovery, *MLFS* morphological leukemia-free state.

Diagnosis and follow-up samples were available for 216/246 patients (88%). At diagnosis, at least one aberrant category affecting ≥10% or ≥5% of the myP/M could be detected in 152/216 (70%) or 179/216 patients (83%), respectively. In the following, the 10% threshold for the presence of a LAIP was used as formerly recommended by ELN [2, 23, 45]. In 56/152 (37%) of patients the aLAIP was defined exclusively by aberrant subpopulations with markers of immaturity and in 72/152 (47%) without markers of immaturity. The most common aberrant category at diagnosis was deficiency of CD13 in 75/152 patients (49%). Cross-lineage expression of CD56, CD7 and deficiency of CD33 was detectable in 44/152 (29%), 44 (29%), and 41 patients (27%), respectively. One, two, or three categories were simultaneously detectable in 103/152 (68%), 46 (30%), and 3 (2%), with the most common combination of CD13 deficiency plus cross-lineage expression of CD56 in 21/49 (43%) of the patients.

**Fig. 2:**
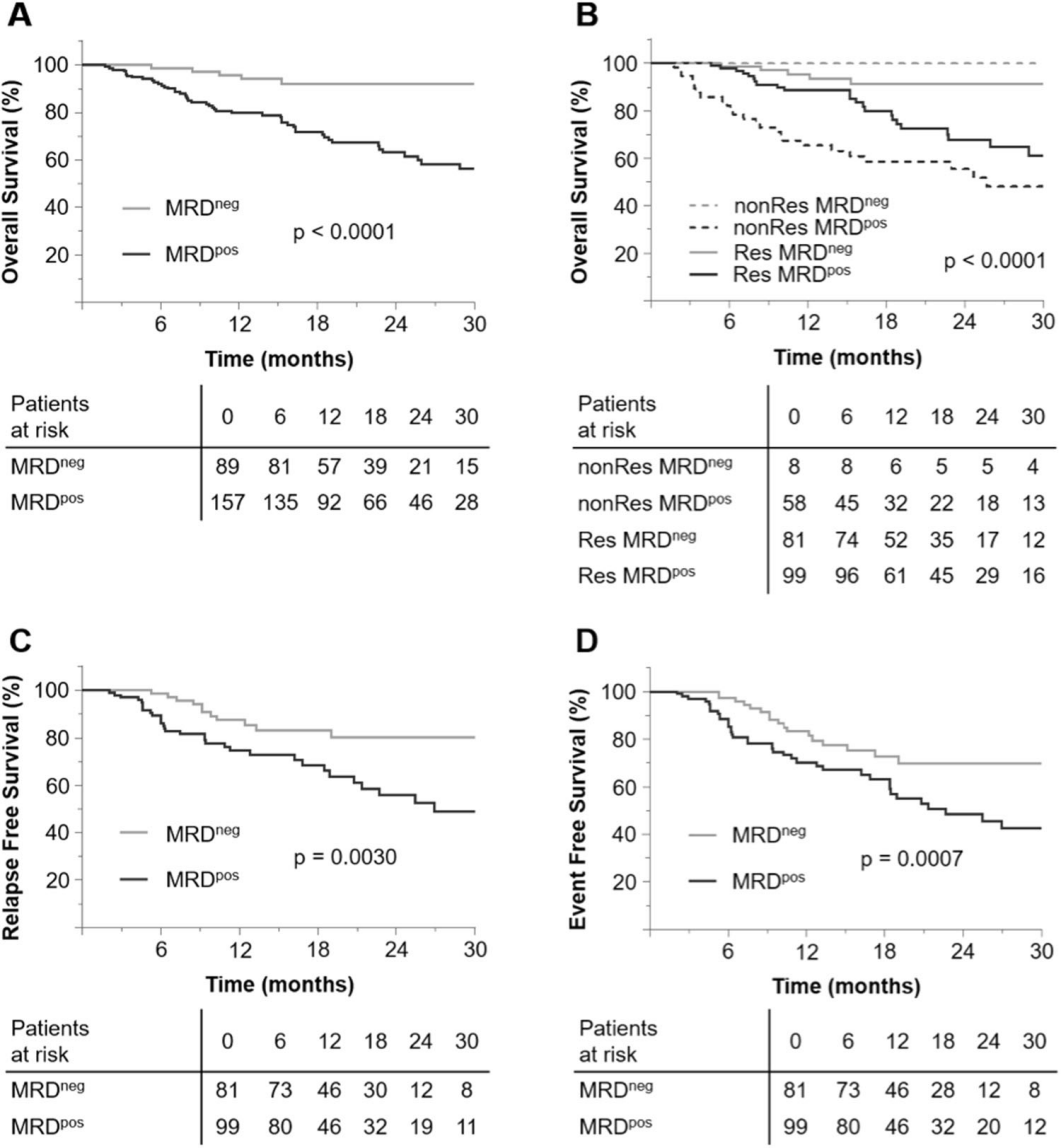
Outcome stratified by the proposed LAIP-based DfN MRD approach. **A** Overall survival for all patients, **B** Overall survival stratified by cytomorphologic response and MRD status, **C** Relapse free survival, **D** Event free survival.

At follow-up, in total 157/246 patients (64%) were MRD^pos^ (Fig. 2A). They were classified as MRD^pos^ by aLAIP^only^, ptDfN^only^ and aLAIP/ ptDfN in 33/157 (21%), 80 (51%) and 44 (28%) cases, respectively. In MRD^pos^ patients, deficiency of CD13 or CD33, cross-lineage expression of CD7 or CD56 was observed in 76/157 (48%), 66 (42%), 74 (47%) and 87 (55%). One, two, three and four aberrant categories could be simultaneously detected in 71/157 (45%), 41 (26%), 30 (19%), and 15 (10%). Most of the patients of the MRD^pos^ group had undergone immunophenotyping at diagnosis (137/157; 87%).

In the subgroup of responders (*n* = 180) the proportion of MRD^pos^ patients was significantly lower compared to non-responders (*n* = 66): 99/180 (55%) versus 58/66 (89%; *p* < 0.0001). For most of the MRD^pos^ responders a measurement at diagnosis (86/99; 87%) was available and MRD^pos^ was classified by aLAIP^only^, ptDfN^only^ and aLAIP/ ptDfN in 24/86 (28%), 45 (52%) and 17 (20%), respectively (Fig. 2B).

MRD assessment by three independent investigators for a cohort of 117 consecutive samples showed that 107/117 (92%) cases were classified concordantly leading to a Kα of 0.86.

The median follow-up time for the entire cohort was 18.9 months (IQR 10.9–29). Compared to MRD^neg^ patients, the OS was significantly shorter in the MRD^pos^ group (HR 5.6, CI: 2.2–14.1, Fig. 2A). Thus, the 2-year OS was 92% (CI: 86–99%) for MRD^neg^ and 63% (CI: 55–73%) for MRD^pos^, respectively. MRD status retained its importance on OS in patient cohorts stratified according to response (CR/CRi/MLFS and RD/PR, Fig. 2B). In responders, the 2-year OS was 91% (CI: 84–99%) for MRD^neg^ and 68% (CI: 57–81%) for MRD^pos^ (HR 3.8; CI: 1.5–10.0; *p* = 0.006). The OS for the MRD^neg^ non-responders was comparable with MRD^neg^ responders. The MRD status also retained its impact on OS after stratifying patients according to ELN risk category (Supplementary Fig. 1A–C).

Regarding RFS and EFS, MRD^pos^ was associated with a significantly shorter RFS and EFS (HR 2.6, CI: 1.4–5.0; and HR 2.7, CI: 1.5–5.0, Fig. 2C, D).

Most importantly, in a multivariable Cox regression model, MRD^pos^ retained its significant prognostic impact on OS for all patients, OS for responders, RFS and EFS (Table 3).

**Table 3:** Multivariable Cox regression model of the proposed MRD approach; ELN 2017: ordinal scale; age: rational scale; ECOG: ordinal scale; sAML: nominal scale; tAML: nominal scale.

	OS – all patients	OS - responder	RFS	EFS
	HR (CI; *p*-value)	HR (CI; *p*-value)	HR (CI; *p*-value)	HR (CI; *p*-value)
MRD^pos^	3.7 (1.4–9.5; 0.007)	3.0 (1.1–8.6; 0.036)	2.1 (1.0–4.3; 0.047)	2.3 (1.2–4.5; 0.018)
ELN fav	1.3 (0.7–2.5; 0.410)	1.1 (0.4–2.8; 0.841)	1.0 (0.5–2.0; 0.996)	0.8 (0.4–1.5; 0.458)
ELN adv	0.6 (0.3–1.4; 0.251)	1.7 (0.7–4.4; 0.275)	1.1 (0.5–2.3; 0.786)	0.9 (0.5–1.9; 0.846)
age	1.0 (1.0–1.1; 0.134)	1.0 (1.0–1.0; 0.906)	1.0 (1.0–1.0; 0.878)	1.0 (1.0–1.0; 0.739)
ECOG 2–4	2.1 (0.8–5.4; 0.128)	1.0 (0.2–4.3; 0.993)	1.3 (0.5–3.7; 0.645)	2.6 (1.1–6.3; 0.034)
sAML	1.0 (0.4–2.4; 0.988)	0.6 (0.1–4.4; 0.590)	0.3 (0.0–2.3; 0.249)	0.3 (0.0–1.9; 0.182)
tAML	1.1 (0.5–2.7; 0.795)	2.0 (0.7–5.5; 0.176)	1.7 (0.7–4.1; 0.210)	1.8 (0.8–4.2; 0.155)

^a^*OS* overall survival, *RFS* relapse free survival, *EFS* event free survival, *HR* hazard ratio, *CI* confidence interval, *ELN fav/ adv* favorable/ adverse risk according to European LeukemiaNet risk stratification by genetics 2017, *ECOG* Eastern Cooperative Oncology Group Performance Status Scale, *sAML* secondary AML, *tAML* therapy related AML.

As ELN recommends a 0.1% cut-off [29], we interpreted our data in two additional ways. First, the analysis was restricted to aberrant subpopulations expressing at least one marker of immaturity (CD34^+^ and/or CD117^+^) and the appendant reference values were uniformly set to 0.1%. In this context, evidence of MRD was termed MRD^posELN^. Of the 246 patients, only 24% (*n* = 60) fulfilled MRD^posELN^ criteria. The MRD^ELN^ analysis still showed significant prognostic relevance, however, the discriminatory power was less compared to the original strategy (Supplementary Fig. 2A). Second, MRD^pos^ was subdivided according to the MRD load in MRD^posLo^ (the reference value was exceeded by <0.1) and MRD^posHi^ (excess of the reference value by ≥0.1). Of the 157 MRD^pos^ patients, one third was assigned to the MRD^posLo^ cohort. The MRD load (MRD^posLo^ vs. MRD^posHi^) did not provide further prognostic information regarding OS (Supplementary Fig. 2B).

As many analysis strategies are focused on aberrancies in the immature compartment, the data was further analyzed utilizing a strategy restricted to aberrant subpopulations expressing at least one of those markers (24 subpopulations) and compared to the strategy encompassing all aberrant subpopulations (32 subpopulations). Of the 246 patients, 49% (*n* = 121) were classified as MRD^posImmOnly^ providing slightly less prognostic impact compared to the proposed MRD approach utilizing all subpopulations. Differential analysis of MRD^pos^ by aLAIP^only^, ptDfN^only^ or aLAIP/ ptDfN did not improve the prediction of outcome (Supplementary Fig. 2C, D, Table 5).

With respect to clinical characteristics (Table 2), variables known to negatively impact patient outcome were enriched in MRD^pos^ patients (age, ELN risk category, karyotype, *FLT3* mutation status, MRC score and morphological response). Accordingly, the frequency of aHSCT was higher in the MRD^pos^ group (MRD^neg^ 55% (49/89) versus MRD^pos^ 77% (121/157), *p* < 0.0001).

### Cross-validation

The convLAIP approach was applicable to *n* = 106 cases with measurements at diagnosis and follow-up. In 99% of the pre-therapeutic samples at least one traceable aLAIP could be detected by the convLAIP approach, in contrast to 78% by the proposed methodology. The aLAIP detected by both approaches typically shared similar features (in 99% of cases). Kα for MRD assessment by convLAIP was 0.59. The MRD status of 69% of follow up samples was rated concordantly by the convLAIP approach and the proposed approach (convLAIP^pos^/ MRD^pos^ 39%, convLAIP^neg^/ MRD^neg^ 30%). There was disagreement on the MRD status in 31% of follow up samples, almost always as convLAIP^neg^/MRD^pos^ constellation (Supplementary Table 4). Nevertheless, the convLAIP provided prognostic power regarding overall survival (Supplementary Fig. 3A), but convLAIP^neg^/ MRD^pos^ patients showed a comparable outcome to patients rated MRD positive by both approaches (convLAIP^pos^/MRD^pos^, Supplementary Fig. 3B).

The Unsup pipeline was applicable to 244/246 (99%) of follow-up measurements. The Unsup pipeline and the proposed approach showed a slightly higher concordance on MRD rating (Unsup^pos^/ MRD^pos^ 45%, Unsup^neg^/ MRD^neg^ 28%) compared to the conventional LAIP approach. This time, inconsistent results were spread to both conflicting categories (Unsup^neg^/ MRD^pos^ 19%, Unsup^pos^/ MRD^neg^ 8%) (Supplementary Table 4). The Unsup pipeline also provided significant prognostic power (Supplementary Fig. 3C).

In 99/246 patients (40%), decisive molecular markers for MRD monitoring were available at diagnosis (m*NPM1*: 78, *RUNX1::RUNX1T1*: 8, *CBFB::MYH11*: 13). For 85 of these 99 patients (m*NPM1*: 67, *RUNX1::RUNX1T1*: 6, *CBFB::MYH11*: 12) molecular MRD results at follow-up were available. In addition, tracking of less established molecular markers (biallelic m*CEBPA:* 7, m*CEBPA-TAT*: 1, m*DNMT3A*: 1, *FLT3-ITD*: 4, *FLT3-TKD*: 1, m*IDH1*: 1, m*IDH2*: 7, *KMT2A::MLLT3*: 2, *KMT2A-PTD*: 10, *PICALM::MLLT10*: 1, m*RUNX1*: 2, m*SRSF2*: 1, m*TET2*: 1, m*TP53*: 1, m*UTAF1*: 1) was done. Discordant results (Mol^neg^/ MRD^pos^ or Mol^pos^/ MRD^neg^) were observed in 15/126 (12%) and 26/126 (21%), respectively (Supplementary Table 4). A shorter OS was observed for Mol^pos^ compared to Mol^neg^ without reaching statistical significance (Supplementary Fig. 3D).

As patients classified as MRD^pos^ by ptDfN^only^ were regarded vulnerable to misinterpretation this cohort was analyzed in more detail. A measurement at diagnosis was available in 59/80 (74%) cases. In 43/59 (73%) of these patients, a minor subclone (<10% of myP/M) with identical aberrant category was already detectable at diagnosis (affecting in median 1.9% of myP/M, IQR 4.2%). The reduction of the population size to define an aLAIP at diagnosis considerably lowered the number of patients classified as MRD^pos^ by ptDfN^only^: ≥10%: 59, ≥5%: 49, ≥2.5%: 41, and ≥1%: 30. Only in 16/59 (27%) patients the aberrant category of ptDfN^only^ was not detectable at all at diagnosis (cross-lineage expression of CD56: 10, deficiency of CD13: 1, and deficiency of CD33: 5). For MRD^pos^ by ptDfN^only^ patients a simultaneous molecular MRD testing was available in 11/80 (14%) cases. Concordant results were observed in 64% of these cases (m*NPM1:* 4, *CBFB::MYH11:* 1, m*IDH2*: 1, *FLT3-ITD:* 1). MRD^pos^ by ptDfN^only^ patients were rated convLAIP^pos^ in 31% of the cases. In 13/80 (16%) CR/CRi samples obtained at later time points (during/after conventional consolidation) from patients rated MRD^pos^ by ptDfN^only^ post-induction, the same “de-novo post treatment aberrant category” could be detected again in 62% of cases. A measurement at relapse was available for 14/80 (18%) patients rated MRD^pos^ by ptDfN^only^ at post-induction. The same “de-novo post treatment aberrant category” could be observed in 71% of relapse samples.

## Discussion

Even though MRD assessment by MFC is technically available for the majority of patients with AML, its broad applicability is still hampered due to the lack of standardization. The focus of this work was to develop a robust, fast and reproducible LAIP-based DfN analysis strategy to evaluate MRD by MFC.

The analysis strategy focused separately on two kinds of abnormalities: reduced expression of myeloid antigens and cross-lineage expression of lymphoid antigens [46]. The reduced expression of CD13 and CD33 as part of a LAIP has been variably described in 10–22% and 18–36% of AML cases, respectively [47–49]. Also, the frequency of the cross-lineage expression of CD7 and CD56 varies substantially with 17–43% [45, 49, 50]. This variability is not only explainable by the aberration-defining gate itself, but also preceding gating steps and the reference population have a major impact on the observation frequency.

Deviating from most analysis strategies for MRD-assessment, we decided to establish one single tube, but augmented the number of populations to be analyzed. The progenitor cell gate was expanded to include monocytes (P/M). P/M cells were required to express at least CD13 or CD33 (myP/M). At diagnosis, this myeloid assignment was negligible as in median 94% (IQR 18.1%) of cells in the P/M gate fulfilled this criterion. At follow-up in median only 84% (IQR 20.3%) of P/M cells met this specification. As unique selling point our MRD analysis includes also the CD34^-^CD117^-^ compartment within myP/M largely representing monopoietic cells. Indeed, acute monoblastic/monocytic leukemia represents approximately 12% of the AML patients [51] and shows an expression of CD34^+^ or CD117^+^ only in 7.7% and 19.8% of cases, respectively [37]. Within our approach, the reference values for the aberrant populations without markers of immaturity were higher compared to aberrant populations expressing either CD34 or CD117 (Supplementary Table 5). For other entities like MDS, the evaluation of monocytes using e.g., CD56 is part of various diagnostic scores [52, 53]. In addition, CD56 expression has been described to distinguish clonal monocytes within CMML from reactive monocytosis [54–56]. These observations have led us to also analyze aberrations outside of the CD34^+^CD117^+^ compartment. In fact, exclusion of aberrant populations without expression of markers of immaturity mostly led to a decline in the informative value of the here proposed MRD approach as calculated by the AIC, which supports the assumption that populations beyond phenotypically immature cells also contain prognostic information. Most MRD^pos^ patients (*n* = 84) showed aberrancies in both compartments. Of note, 36 patients were classified as MRD^pos^ solely by aberrations within the compartment without expression of markers of immaturity. Some of these patients were at the same time also HLA-DR negative. The most common aberrant categories were cross-lineage expression of CD56 and deficiency of CD33 (each 44%). A cross-lineage expression of CD7 or a deficiency of CD13 was not found in these cases. This observation fits well with previously published data reporting that leukemic immature monocytes used for MRD monitoring by a LAIP-approach were frequently characterized by decreased expression of HLA-DR and increased expression of CD56 and CD13 [57]. Again, the assignment of these cells to the monopoietic compartment and their maturity remained somewhat speculative as the panel did not allow a proper categorization as neither monocytic markers nor other antigens associated with immaturity e.g., CD133 were evaluated [58].

In only 28% of MRD^pos^ responders, the rating was based solely on the detection of an aberrant category already evident at diagnosis, whereas ptDfN^only^ defined MRD^pos^ in 51% of cases. This unexpected high rate of ptDfN^only^ is partly related to the availability of measurements at diagnosis and the LAIP definition used in our approach. By modifying the aLAIP definition to ≥5% of myP/M, in 83% of patients at least one LAIP was detectable at diagnosis and the MRD^pos^ rate by ptDfN^only^ dropped from 38% to 31%. A minor subclone with identical aberrant category was already detectable at diagnosis in 73% of these patients. In addition, the assumption that ptDfN^only^ mostly represents “true” MRD than phenotypic shifts, was supported by simultaneous molecular MRD testing that showed 64% concordant results. Furthermore, the same “de-novo post treatment aberrant category” could be observed in 71% of relapse samples. Indeed, selection pressure by chemotherapy can change the original composition and initially existing but rather small populations expand, as documented for (molecular) genetics [59] and immunophenotypes [21]. However, phenotypic shifts might have also contributed. This conception was supported by the observation that 32/105 (30%) patients were assessed discordantly at follow-up using the proposed MRD approach and the convLAIP approach (convLAIP^neg^/ MRD^pos^). Features of the aLAIP defined by the convLAIP at diagnosis were detected in 99% by the proposed MRD approach. Leukemic cells can undergo phenotypic shifts during the course of the disease due to an evolution (emergence of not previously present immunophenotypes). At relapse, a gain in the expression of immaturity markers is frequently described [21]. Phenotypic shifts as a result of selection pressure or clonal evolution are usually not clearly distinguishable, but nevertheless representing both “true” MRD^pos^. But abnormalities in immunophenotype have been also observed leukemia-independent (age and treatment related) and might result in false positive results [20, 25, 60]. Clonal hematopoiesis is also suggested to be associated with phenotypic aberrations [61, 62].

The reference values for the aberrant populations with markers of immaturity correspond well with previously published sensitivity levels of MFC methods (10^−4^–10^−5^) [49, 63]. To smoothen the heterogeneity in reference values, ELN recommended a flat 0.1% cut-off (10^−3^) as this level had been of prognostic relevance in most publications and is at least one log above the published sensitivity level for MRD by MFC [63]. In our approach, 7 out of 32 aberrant populations presented with reference values >10^−3^, so these subpopulations (all expressed neither of CD34 nor CD117) had to be excluded from the analysis with this uniform cut-off (termed MRD^posELN^). In the end, the different approaches reduced the informative value of our gating strategy.

The quality of MRD assessment by MFC is considerably affected by the reference values. Although, the cohort of ALL molCR only represented 21% of the LFC samples, roughly this cohort determined 50% of the reference values. BMD, the most commonly used LFC cohort with predominantly young subjects, represented 30% of the LFC cohort and only established 31% of reference values. This distribution pattern underpins the necessity to include a broad range of different LFC cohorts as various factors (e.g., prior exposition to chemotherapy and age) substantially influence the frequency of certain expression profiles.

Most importantly, our LAIP-based DfN analysis strategy (including cell compartments with and without expression of markers of immaturity) provided significant prognostic information on clinical outcome after intensive induction treatment. MRD^pos^ patients showed a significantly shorter OS and a higher relapse risk, both in univariable as well as multivariable regression models. The 2017 ELN genetic risk stratification is frequently used for pretreatment risk assessment [2]. Of note, our MRD results helped to further segregate the prognosis within each ELN risk category. The MRD status was most predictive for outcome in patients with favorable and adverse risk. The importance in the adverse risk category was not surprising, as MRD status pre-transplant has been described to be of prognostic significance [46]. Whether allogeneic transplantation and intensity of the conditioning regimen can have an influence on the MRD-associated prognosis is a matter of debate [39, 64, 65]. The discriminatory power of the MRD status remained valid when established baseline prognostic variables were considered. MRD^pos^ was associated with adverse outcome in responding as well as non-responding patients. However, in non-responders, the MRD status did not reach statistical significance due to low patient numbers. Nevertheless, our data suggest that the proposed approach can reliably distinguish vigorous hematopoietic regeneration with an increase in normal progenitors from persistence of leukemic cells. This is of particular importance as both scenarios are associated with a totally different prognosis. Summarizing, our analysis strategy could confirm the prognostic significance of the MRD status after intensive induction treatment.

In contrast to previous reports, we explicitly focused on applicability of the MRD assessment within clinical routine. Current protocols with manual gating are time-consuming (no published data available), they rely on the expertise of the individual investigator and are therefore prone to inter-rater variations [20, 34]. The proposed MRD approach is fast and shows a very promising IRR. Artificial intelligence is established as a research tool in order to circumvent these disadvantages [39, 66, 67], but has not been implemented as diagnostic test in the daily clinical routine yet. The introduction of fixed gates within our approach resulted in a high inter-rater reliability with respect to both, LFC and AML samples and in short analysis time.

## Conclusion

We present a hierarchical gating strategy, combining the LAIP and DfN analysis approaches, which allows a high level of MFC standardization and a promising inter-rater reliability in MRD detection. Our standardized MFC approach is implementable at other laboratories and enables standardized multicentric immunophenotypic MRD assessment. Such standardization is an important step towards individualized treatment decisions within routine AML therapy and MFC may thus also serve as a biomarker within prospective clinical trials.

## Supplementary information

Supplemental material
