# Supplementary material for: Reproducible measurable residual disease detection by multiparametric flow cytometry in acute myeloid leukemia
Source: Leukemia. 2022 Jul 18;36(9):2208–17. doi: 10.1038/s41375-022-01647-5 (PMC9417981; doi:10.1038/s41375-022-01647-5)
Supplement: Supplementary file 1 — Supplemental material [file 41375_2022_1647_MOESM1_ESM.docx]

## Supplemental material

## Methods

### Sample preparation and acquisition

Cell preparation was performed within 48 h after BM aspiration. Prior to staining, erythrocytes were removed by bulk lysis (10 min at room temperature) using BD Pharm Lyse (1:10 dilution with distilled water; BD Biosciences, San Jose, CA) followed by two washing steps with phosphate-buffered saline (PBS, Life technologies, Carlsbad, CA). For surface labeling, cells were incubated with mAb in the dark (15 min at room temperature). Subsequently, cells were washed twice and resuspended in 500 µl PBS.

Samples were acquired on a BD FACSCanto equipped with three lasers (488 nm, 633 nm, 405 nm) using BD FACSDiVa 9.0 software. Photomultiplier voltages were defined using BD Flow-Set Pro Fluorosphere Beads according to the Harmonemia recommendations^35^ and stored as application settings in the BD FACSDiVa 9.0 software. For compensation, BD CompBeads and the automated compensation tool of the BD FACSDiVa 9.0 software were used. Daily quality controls included a performance check with BD CS&T beads and an 8-color-control using StatusFlow Whole Blood Control (Biozol, Eching, Germany). Application and compensation settings were linked to the experiment template.

### Analytical performance

To further determine the ability of this approach to quantify MRD load, serial dilution assays with 3 AML cell lines (OCI-AML3, HL60, and KG-1; DSMZ, Braunschweig, Germany, Supplemental table 3) and 29 samples from untreated AML were performed. Primary AML samples and AML cell lines were stepwise-diluted using LFC BM aspirates (two-fold dilution steps starting with 1:2). For primary AML samples, the first dilution step was carried out volumetrically. For AML cell lines, dilution with the LFC sample started at equal white blood cell numbers. Cell numbers were determined using a XN-20 hematology analyzer (Sysmex, Kobe, Japan). Depart from the above-described gating strategy, the myP/M gate in AML cell line samples was permanently enlarged to assure the inclusion of the blasts of the cell line but without including the granulopoiesis of the LFC. Diluted samples with any aberrant subpopulation above the value of the undiluted LFC (empty value) were considered as positive.

### MRD load

The MRD load was defined as the aberrant category with the highest percentage of CD45^+^ events. To investigate the influence of the MRD load on patient outcome, we dichotomized the patients according to the main clinical endpoints (survival and relapse) and compared the MRD load among these cohorts. Furthermore, we plotted MRD load against the time until occurrence of these clinical endpoints.

## Results

### Analytical performance

To determine the capability to quantify MRD, dilution experiments were performed using a linear regression model for each aberrant category. For this analysis, the initial size of the aberrant category was normalized to 0.5 for the first dilution step (1:2). For this assumption, a slope of 1.00 would indicate an ideal quantifiability.

The median slope of the linear regression models of the AML cell lines was 0.99 (IQR 0.03) (n=18 measurement). For the aberrant categories deficiency of CD13 or CD33, cross-lineage-expression of CD7 or CD56, the median slope was calculated as 1.03 (IQR 0.18, n=4), 1.02 (IQR 0.02, n=2), 0.98 (IQR 0.06, n=5) and 0.99 (IQR 0.02, n=2), respectively.

The median slope of the primary AML samples was 0.99 (IQR 0.10) (n=64 measurements). For the aberrant categories deficiency of CD13 or CD33, cross-lineage expression of CD7 or CD56 the median slope was 0.98 (IQR 0.21, n=16), 0.97 (IQR 0.10, n=11), 1.02 (IQR 0.10, n=18) and 0.98 (IQR 0.06, n=8), respectively.

In AML cell lines and primary AML samples the 95% confidence interval of the slope of the linear regression model always included the ideal slope of 1.

### MRD load

MRD load of surviving patients (median 0.31%, IQR 1.66%) was not statistically different to the MRD load of those patients who died during the observation period (median 0.78%, IQR 3.17%; p=0.1525, Supplemental Figure 4A). We also observed no statistically significant difference when MRD positive patients were dichotomized by no‑relapse/relapse (no-relapse: median 0.14%, IQR 0.51%; relapse: median 0.15%, IQR 0.80%, p=0.2322, Supplemental Figure 4B). In addition, the time to event (overall survival and relapse free survival) did not correlate with MRD load (Supplemental Figure 4C/D).

### Supplemental tables

| **All leukemia-free controls** | |  |  |
| --- | --- | --- | --- |
| **n=90** | |  |  |
| **Age [years]** | | Median (IQR) | 63 (40) |
| **Gender [%]** | | female | 35 |
|  |  | male | 37 |
|  |  | missing | 17 |
|  | **Bone marrow donors** |  |  |
|  | **n=30** |  |  |
|  | **Age [years]** | Median (IQR) | 30 (11) |
|  | **Gender [%]** | female | 4 |
|  |  | male | 10 |
|  |  | missing | 16 |
|  | **Primary central nervous system lymphoma** | | |
|  | **n=9** |  |  |
|  | **Age [years]** | Median (IQR) | 62 (15) |
|  | **Gender [%]** | female | 4 |
|  |  | male | 5 |
|  |  | missing | 0 |
|  | **ALL in molecular complete remission** | | |
|  | **n=19** |  |  |
|  | **Age [years]** | Median (IQR) | 47 (35) |
|  | **Gender [%]** | female | 5 |
|  |  | male | 12 |
|  |  | missing | 0 |
|  | **Bone marrow from hip surgery** | | |
|  | **n=19** |  |  |
|  | **Age [years]** | Median (IQR) | 75 (8) |
|  | **Gender [%]** | female | 22 |
|  |  | male | 10 |
|  |  | missing | 0 |

Supplemental Table 1: Clinical characteristics of the leukemia-free controls. *IQR* interquartile range, *ALL* acute lymphoblastic leukemia

| **Fluorochrome** | **Antigen** | **Clone** |
| --- | --- | --- |
| **FITC** | CD34 * | 8G12 |
| **PE** | CD13 * | L138 |
| **PerCP-Cy5.5** | CD7 * | M-T701 |
| **PE-Cy7** | CD33 ^#^ | D3HL60.251 |
| **APC** | CD56 * | NCAM16.2 |
| **AlexaFluor750** | CD117 ^#^ | 104D2D1 |
| **PacBlue** | HLA DR ^#^ | Immu357 |
| **V500** | CD45 * | HI30 |

Supplemental Table 2: Monoclonal antibody panel (*BD Bioscience; ^#^Beckman Coulter)

| **Myeloid progenitor/monocyte (myP/M) main populations** | | | | | | | | |  |  |
| --- | --- | --- | --- | --- | --- | --- | --- | --- | --- | --- |
| **CD34** | | **CD117** | | | **HLA-DR** | | **Reference value** | **Krippendorffs α** | **reference value defining LFC cohort** | **MRD^pos^ AML cases**  [%] |
|  |  |  |  |  |  |  | [% of CD45^+^] |  |  |  |
| **+** | | **+** | | | **+** | | 0.566 | 0.986 | Not applied for MRD | Not applied for MRD |
|  |  |  |  |  | **-** | | 0.107 | 0.972 | Not applied for MRD | Not applied for MRD |
|  |  | **-** | | | **+** | | 0.456 | 0.979 | Not applied for MRD | Not applied for MRD |
|  |  |  |  |  | **-** | | 0.184 | 0.958 | Not applied for MRD | Not applied for MRD |
| **-** | | **+** | | | **+** | | 0.431 | 0.925 | Not applied for MRD | Not applied for MRD |
|  |  |  |  |  | **-** | | 0.174 | 0.917 | Not applied for MRD | Not applied for MRD |
|  |  | **-** | | | **+** | | 5.333 | 0.982 | Not applied for MRD | Not applied for MRD |
|  |  |  |  |  | **-** | | 7.162 | 0.959 | Not applied for MRD | Not applied for MRD |
|  |  | | | | | | | |  |  |
|  | **Deficiency of CD13 within subpopulations** | | | | | | | |  |  |
|  | **CD34** | | **CD117** | **HLA-DR** | | **CD13** | **Reference value** | **Krippendorffs α** | **reference value defining LFC cohort** | **MRD^pos^ AML cases**  [%] |
|  |  |  |  |  |  |  | [% of CD45^+^] |  |  |  |
|  | **+** | | **+** | **+** | | **-** | 0.008 | 0.967 | ALL | 16 |
|  |  |  |  | **-** | | **-** | 0.003 | 0.990 | ALL | 9 |
|  |  |  | **-** | **+** | | **-** | 0.014 | 0.971 | ALL | 12 |
|  |  |  |  | **-** | | **-** | 0.005 | 0.980 | ALL | 13 |
|  | **-** | | **+** | **+** | | **-** | 0.011 | 0.843 | ALL | 24 |
|  |  |  |  | **-** | | **-** | 0.011 | 0.757 | ALL | 36 |
|  |  |  | **-** | **+** | | **-** | 1.992 | 0.871 | ALL | 4 |
|  |  |  |  | **-** | | **-** | 1.588 | 0.922 | ALL | 8 |
|  |  | | | | | | | |  |  |
|  | **Deficiency of CD33 within subpopulations** | | | | | | | |  |  |
|  | **CD34** | | **CD117** | **HLA-DR** | | **CD33** | **Reference value** | **Krippendorffs α** | **reference value defining LFC cohort** | **MRD^pos^ AML cases**  [%] |
|  |  |  |  |  |  |  | [% of CD45^+^] |  |  |  |
|  | **+** | | **+** | **+** | | **-** | 0.048 | 0.983 | Hip surgery | 6 |
|  |  |  |  | **-** | | **-** | 0.032 | 0.985 | BMD | 5 |
|  |  |  | **-** | **+** | | **-** | 0.043 | 0.976 | BMD | 10 |
|  |  |  |  | **-** | | **-** | 0.077 | 0.992 | PCNSL | 10 |
|  | **-** | | **+** | **+** | | **-** | 0.008 | 0.863 | PCNSL | 10 |
|  |  |  |  | **-** | | **-** | 0.006 | 0.849 | BMD | 15 |
|  |  |  | **-** | **+** | | **-** | 0.028 | 0.934 | BMD | 16 |
|  |  |  |  | **-** | | **-** | 0.138 | 0.822 | Hip surgery | 30 |
|  |  |  |  |  |  |  |  |  |  |  |

|  | **Cross-lineage expression of CD7 within subpopulations** | | | | | |  |  |
| --- | --- | --- | --- | --- | --- | --- | --- | --- |
|  | **CD34** | **CD117** | **HLA-DR** | **CD7** | **Reference value** | **Krippendorffs α** | **reference value defining LFC cohort** | **MRD^pos^ AML cases**  [%] |
|  |  |  |  |  | [% of CD45^+^] |  |  |  |
|  | **+** | **+** | **+** | **+** | 0.012 | 0.802 | ALL | 28 |
|  |  |  | **-** | **+** | 0.002 | 0.867 | BMD | 20 |
|  |  | **-** | **+** | **+** | 0.019 | 0.767 | BMD | 21 |
|  |  |  | **-** | **+** | 0.005 | 0.718 | ALL | 31 |
|  | **-** | **+** | **+** | **+** | 0.047 | 0.854 | BMD | 15 |
|  |  |  | **-** | **+** | 0.012 | 0.793 | Hip surgery | 26 |
|  |  | **-** | **+** | **+** | 0.593 | 0.928 | Hip surgery | 5 |
|  |  |  | **-** | **+** | 0.119 | 0.885 | ALL | 17 |
|  |  | | | | | |  |  |
|  | **Cross-lineage expression of CD56 within subpopulations** | | | | | |  |  |
|  | **CD34** | **CD117** | **HLA-DR** | **CD56** | **Reference value** | **Krippendorffs α** | **reference value defining LFC cohort** | **MRD^pos^ AML cases**  [%] |
|  |  |  |  |  | [% of CD45^+^] |  |  |  |
|  | **+** | **+** | **+** | **+** | 0.010 | 0.791 | BMD | 25 |
|  |  |  | **-** | **+** | 0.001 | 0.630 | BMD | 11 |
|  |  | **-** | **+** | **+** | 0.010 | 0.767 | ALL | 20 |
|  |  |  | **-** | **+** | 0.003 | 0.751 | BMD | 22 |
|  | **-** | **+** | **+** | **+** | 0.023 | 0.835 | ALL | 23 |
|  |  |  | **-** | **+** | 0.013 | 0.726 | Hip surgery | 25 |
|  |  | **-** | **+** | **+** | 0.637 | 0.953 | ALL | 32 |
|  |  |  | **-** | **+** | 1.045 | 0.941 | ALL | 25 |

Supplemental Table 3: Reference values for aberrant subpopulations as determined in leukemia-free controls (LFC). Krippendorffs α refers to the assessment of three independent investigators. The cohort of LFC responsible for the definition of the reference value is given in the penultimate column. In the last column the percentage of AML cases with excess of aberrant subpopulation is given. *LFC* leukemia free control, *MRD* measurable residual disease, *AML* acute myeloid leukemia, *ALL* acute lymphoblastic leukemia, *BMD* bone marrow donor, *PCNSL* primary central nervous system lymphoma

|  | **MRD^pos^** | **MRD^neg^** |
| --- | --- | --- |
| **convLAIP^pos^** | 41/105 (39%) | 1/105 (<1%) |
| **convLAIP^neg^** | 32/105 (30%) | 31/105 (30%) |
|  |  |  |
| **Unsup^pos^** | 111/244 (45%) | 19/244 (8%) |
| **Unsup^neg^** | 46/244 (19%) | 68/244 (28%) |
|  |  |  |
| **Mol^pos^** | 50/126 (40%) | 26/126 (21%) |
| **Mol^neg^** | 15/126 (12%) | 35/126 (28%) |

Supplemental Table 4: Cross-validation of MRD status assessed by various approaches. MRD measurable residual disease, LAIP leukemia associated immunophenotype

|  | **OS – all patients** | **OS - responder** | **RFS** | **EFS** |
| --- | --- | --- | --- | --- |
|  | AIC value (n) | AIC value (n) | AIC value (n) | AIC value (n) |
|  | HR (CI; p-value) | HR (CI; p-value) | HR (CI; p-value) | HR (CI; p-value) |
|  | MRD^pos^ vs. MRD^neg^ | MRD^pos^ vs. MRD^neg^ | MRD^pos^ vs. MRD^neg^ | MRD^pos^ vs. MRD^neg^ |
| **analysis^ImmOnly^** | 531.46 (246) | 266.16 (179) | 435.73 (180) | 496.55 (180) |
|  | 4.5 (2.3-8.9; <0.0001) | 2.7 (1.3-5.8; 0.011) | 2.2 (1.2-3.8; 0.009) | 2.2 (1.3-3.7; 0.004) |
|  | n=121 vs. 125 | n=67 vs. 112 | n=68 vs. 112 | n=68 vs. 112 |
| **analysis^compl^** | 533.81 (246) | 263.26 (179) | 433.34 (180) | 492.56 (180) |
|  | 5.6 (2.2-14.1; <0.0001) | 3.8 (1.5-10.0; 0.006) | 2.6 (1.4-5.0; 0.004) | 2.7 (1.5-5.0; 0.001) |
|  | n=157 vs. 89 | n=98 vs. 8 | n=99 vs. 81 | n=99 vs. 81 |

Supplemental Table 5: AIC values and univariable Cox regression model comparing analysis^ImmOnly^ (only immunophenotypes with markers of immaturity CD34^+^ and/or CD117^+^) and analysis^compl^ (all immunophenotypes). *OS* overall survival, *RFS* relapse free survival, *EFS* event free survival, *AIC* Akaike information criterion, *HR* hazard ratio, *CI* confidence interval, *MRD* measurable residual disease

| **Cell line** | **Immunophenotype** | **MRD category** |
| --- | --- | --- |
| **OCI-AML3** | CD34^-^  CD117^+^  HLA-DR^-^  CD7^-^  CD56^+^  CD13^+^  CD33^+^ | deficiency of CD33  cross-lineage expression of CD7  cross-lineage expression of CD56 |
| **KG-1** | CD34^+^  CD117^+^  HLA-DR^-^  CD7^+^  CD56^+^  CD13^+^  CD33^+^ | cross-lineage expression of CD7  cross-lineage expression of CD56 |
| **HL-60** | CD34^-^  CD117^+^  HLA-DR^-^  CD7^-^  CD56^+^  CD13^+^  CD33^var^ | deficiency of CD13 |

Supplemental Table 6: Characteristics of the utilized AML cell lines,
-, expressed by <10% of myP/M;
var, expressed by 10-50% of myP/M;
+, expressed by >50% of myP/M

### Supplemental figures


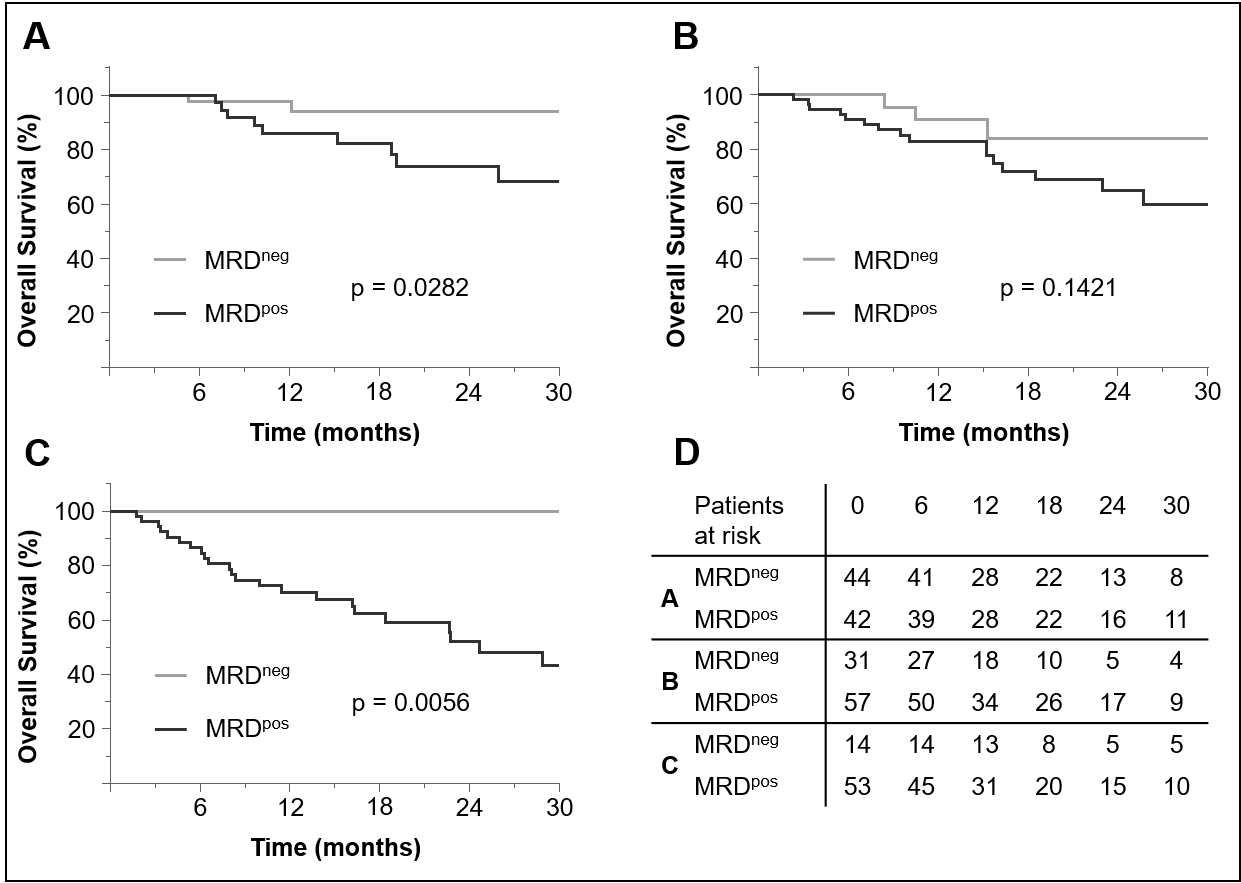


Supplemental Figure 1: OS stratified by ELN risk category. MRD status determined by the proposed MRD approach. (A) favorable, (B) intermediate, (C) adverse risk category according ELN 2017, (D) absolute numbers of patient at risk for each risk category


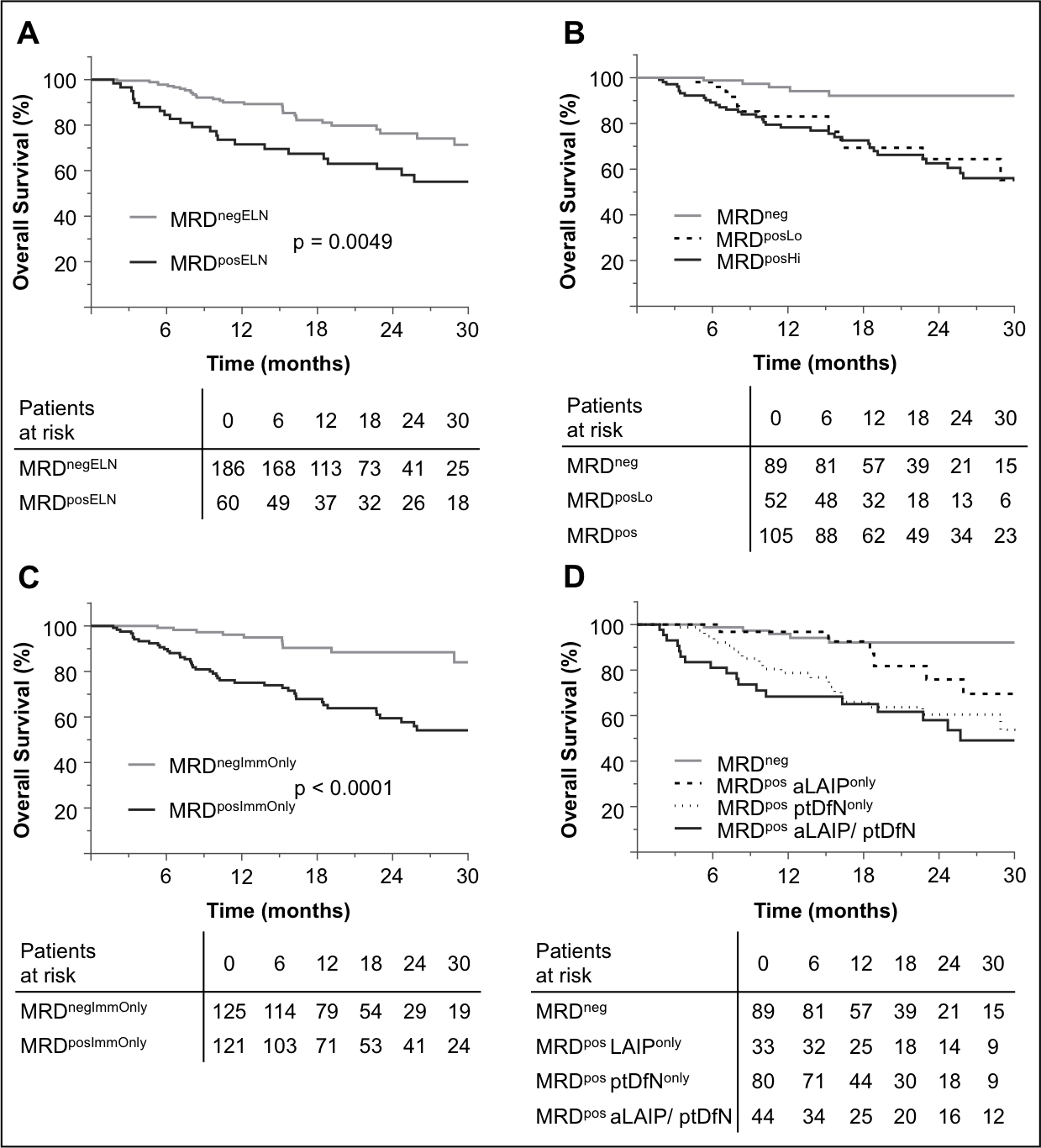


Supplemental Figure 2 Overall survival (A) stratified by a simplified, universal 0.1% MRD cut-off: MRD^negELN^ and MRD^posELN^, (B) stratified by residual disease burden: MRD^neg^, MRD^posLo^ and MRD^posHI^, (C) stratified by only immunophenotypes with markers of immaturity (CD34^+^ and/or CD117^+^): MRD^negImmOnly^ and MRD^posImmOnly^, (D) stratified by the kind of MRD: MRD^neg^, MRD^pos^ by aLAIP^only^, ptDfN^only^ and aLAIP/ ptDfN.


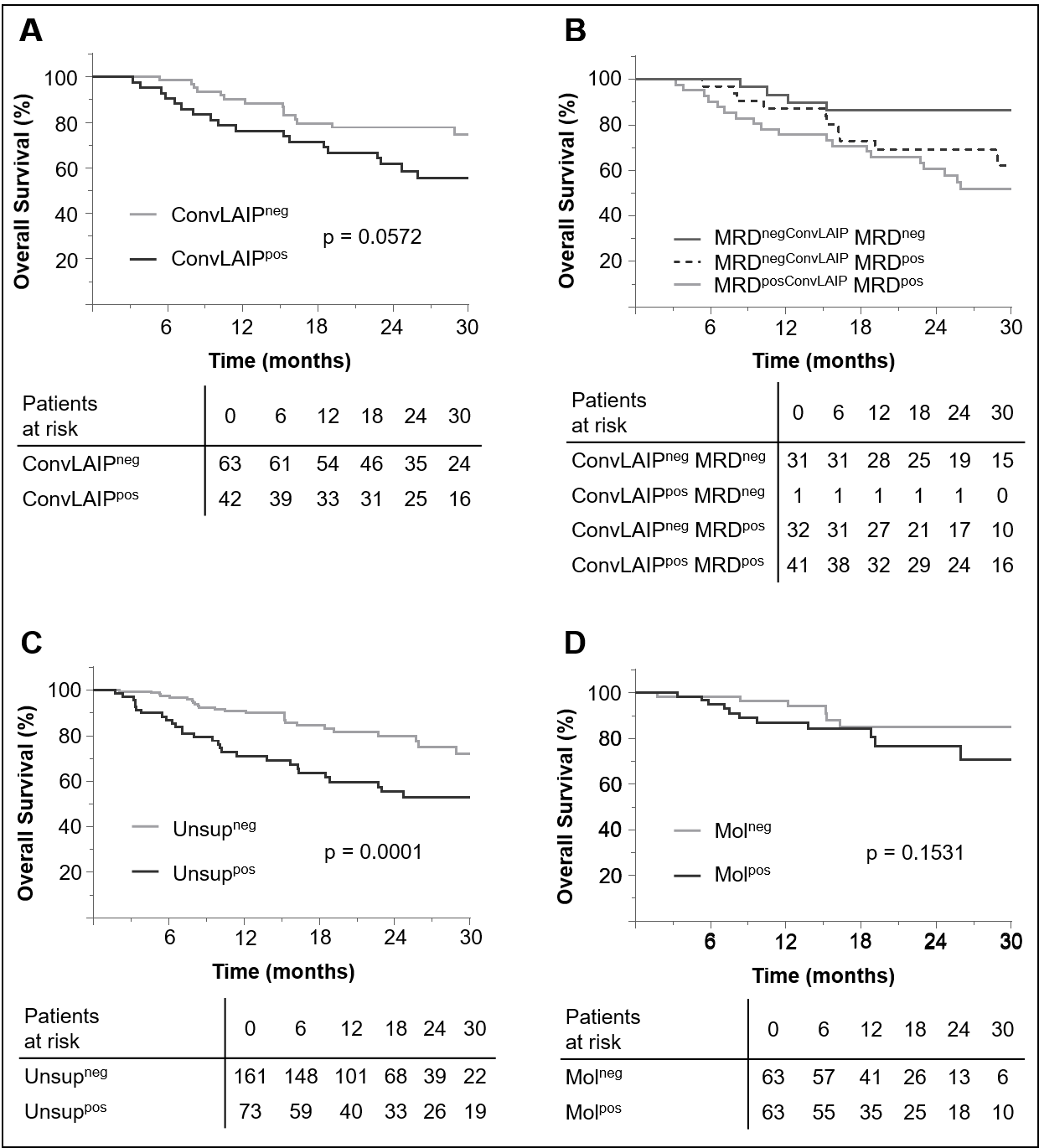


Supplemental Figure 3: OS stratified by various MRD assessment approaches (A) conventional LAIP approach, (B) unsupervised clustering MRD pipeline, (C) MRD status by available molecular markers, (D) combination of the conventional LAIP approach and the proposed MRD approach.


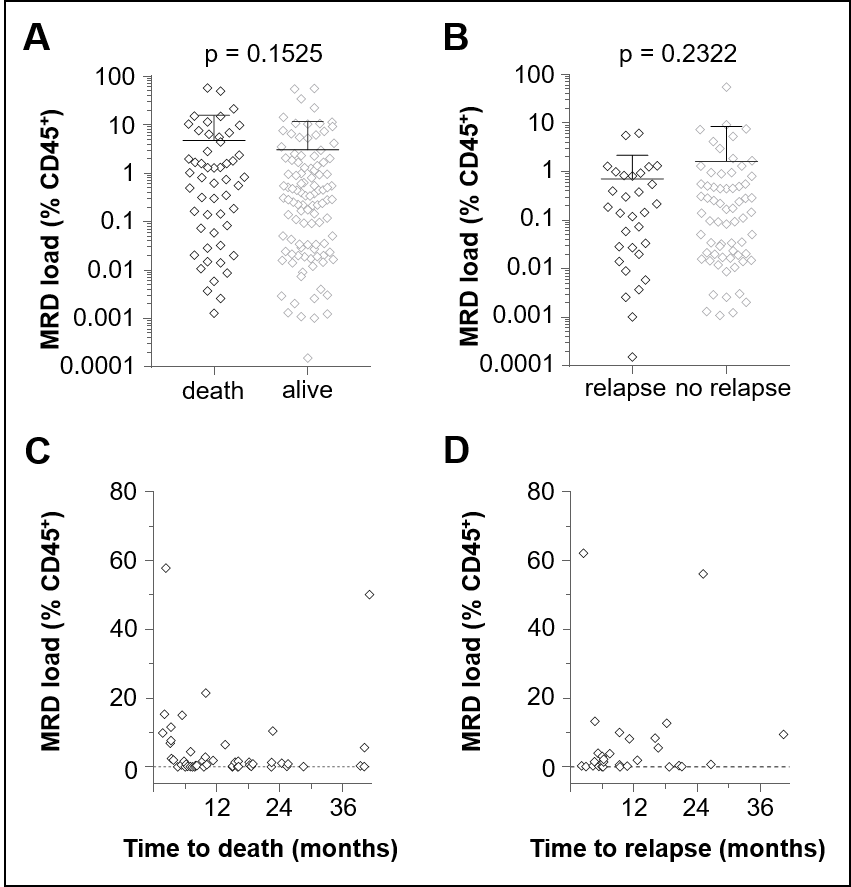


Supplemental Figure 4: MRD load in percentage of CD45^+^ events. (A) MRD^pos^ patients dichotomized by overall survival. (B) MRD^pos^ patients dichotomized by relapse. (C) MRD load plotted against time to death (D) MRD load plotted against time to relapse.
